# Catastrophic Decline of World's Largest Primate: 80% Loss of Grauer's Gorilla (*Gorilla beringei graueri*) Population Justifies Critically Endangered Status

**DOI:** 10.1371/journal.pone.0162697

**Published:** 2016-10-19

**Authors:** Andrew J. Plumptre, Stuart Nixon, Deo K. Kujirakwinja, Ghislain Vieilledent, Rob Critchlow, Elizabeth A. Williamson, Radar Nishuli, Andrew E. Kirkby, Jefferson S. Hall

**Affiliations:** 1 Wildlife Conservation Society, 2300 Southern Boulevard, Bronx, New York 10460, United States of America; 2 Conservation Science Group, Department of Zoology, David Attenborough Building, Cambridge University, Pembroke Road, Cambridge, United Kingdom; 3 North of England Zoological Society, Chester Zoo, Upton by Chester, CH2 1LH, United Kingdom; 4 CIRAD, UPR Forêts et Sociétés, F-34398 Montpellier, France; 5 Department of Biology, University of York, York, United Kingdom; 6 Faculty of Natural Sciences, University of Stirling, Stirling, Scotland, United Kingdom; 7 Institut Congolais pour la Conservation de la Nature (ICCN), Bukavu, Democratic Republic of Congo; 8 Smithsonian Tropical Research Institute, Av. Roosevelt 401, Balboa, Ancon, Panama; University of Lleida, SPAIN

## Abstract

Grauer’s gorilla (*Gorilla beringei graueri*), the World’s largest primate, is confined to eastern Democratic Republic of Congo (DRC) and is threatened by civil war and insecurity. During the war, armed groups in mining camps relied on hunting bushmeat, including gorillas. Insecurity and the presence of several militia groups across Grauer’s gorilla’s range made it very difficult to assess their population size. Here we use a novel method that enables rigorous assessment of local community and ranger-collected data on gorilla occupancy to evaluate the impacts of civil war on Grauer’s gorilla, which prior to the war was estimated to number 16,900 individuals. We show that gorilla numbers in their stronghold of Kahuzi-Biega National Park have declined by 87%. Encounter rate data of gorilla nests at 10 sites across its range indicate declines of 82–100% at six of these sites. Spatial occupancy analysis identifies three key areas as the most critical sites for the remaining populations of this ape and that the range of this taxon is around 19,700 km^2^. We estimate that only 3,800 Grauer’s gorillas remain in the wild, a 77% decline in one generation, justifying its elevation to Critically Endangered status on the IUCN Red List of Threatened Species.

## Introduction

Grauer’s gorilla, together with the mountain gorilla (*Gorilla beringei beringei*), are two subspecies of eastern gorilla (*Gorilla beringei*). Although mountain gorillas are classified as Critically Endangered by IUCN, Grauer’s gorillas have been classified as Endangered [1]. Mountain gorillas have been surveyed regularly since the late 1970s and their small numbers are currently increasing [2–3]; in contrast, there have been few surveys of Grauer’s gorilla and only one attempt to measure population numbers across its range [4–5]. Those surveys, made in 1994–95, used line transects, complete nest counts and reconnaissance surveys to produce an estimate of 16,900 individuals. The 1994–95 surveys did not identify gorilla populations in the Tayna-Usala region; however, which would have increased the total estimate by 1,000–2,000 individuals [6].

The Rwandan genocide in 1994 caused hundreds of thousands of refugees to flee to the DRC and this in turn led to the DRC civil war in 1996 [7], which continued until 2003 with devastating consequences, including an estimated five million people killed, increased insecurity, heightened illegal bushmeat trade and increased deforestation [8]. In eastern DRC, the civil war resulted in the formation of many armed groups, including those born among local communities protecting their interests from other armed groups (*Mai Mai* militia), particularly over access to mining sites [9]. Artisanal mining expanded in North and South Kivu provinces, with most mines controlled by armed militia or soldiers from the national army [10]. Artisanal miners and militia often operate in remote forests, far from villages, and resort to hunting the local fauna to feed themselves, targeting the larger species that provide more meat [11]. Despite being protected by law, gorillas are highly prized as bushmeat because of their large size and they are killed relatively easily with guns since they move in groups on the ground [12] and can be tracked more easily than other large primates, such as chimpanzees (*Pan troglodytes*).

Concern over the status of Grauer’s gorilla has been mounting as the insecurity and inability to implement the rule of law in eastern DRC continue. Predictions of large declines in numbers have been made with limited data, available from only a few locations [6], making it difficult to extrapolate across the Grauer’s range. Here we present an assessment of the current status of Grauer’s gorilla and show that populations have declined drastically across most of its former range.

The 1994–95 surveys estimated that Grauer’s gorillas in Kahuzi-Biega National Park (KBNP), including Kasese to the west, formed 86% of the subspecies total population with the highest densities inside the park [4], and that gorillas in KBNP numbered 7,670 individuals [5]. Using two newly-compiled datasets, we compared 1) density estimates of Grauer’s gorillas in KBNP and adjacent areas, and 2) encounter rates of gorilla nests from 10 sites across its range: Balala Forest; Itombwe Reserve; KBNP (Tshivanga, Nzovu, Itebero combined with the north of Lulingu, and Kasese sectors); Maiko National Park; two sites in the Reserve des Gorilles de Punia (RGPU; Kasese region); and Usala Forest. We then developed an occupancy model with spatial auto-correlation in a hierarchical Bayesian framework to estimate the occupancy probability of gorillas across their range using data collected by park rangers and local community teams who can access the forest even where security is poor. Species range and numbers of gorillas are estimated using this model by identifying a threshold probability, maximizing model performance in predicting gorilla presence and absence.

## Methods

### Data

Three types of data were available that recorded gorilla signs with georeferenced locations: 1) transect data from surveys made in the lowland sector of KBNP between 2011 and 2015; 2) data from reconnaissance walks (recces) to survey presence of gorillas between 2011 and 2015, including visits to specific cells to collect occupancy data; 3) data from patrols made by rangers (in parks) and by local community ecoguards (in community reserves) between 2011 and 2015 and which were stored in freely-available SMART software (Spatial Monitoring and Reporting Tool - www.smartconservationsoftware.org). SMART is a new and improved tool for measuring, evaluating and tracking the effectiveness of wildlife law-enforcement patrols and site-based conservation activities. Recces involve walking in a specific compass direction, but minimising cutting of vegetation by following animal trails or paths. GPS location data were taken at least every 250 metres along transects and at a maximum of every 30 minutes on recces or patrols. Wherever sightings of gorillas, or gorilla sign (nests, trails or feeding sign) were observed, a GPS location was also recorded. JSH and EAW provided data on nest counts from the 1994 transect surveys of Grauer’s gorilla in KBNP. Transect data included perpendicular distance measurements to gorilla nests to enable detection probabilities to be calculated using Distance 6.0 [13].

### Data analysis

#### Density estimates of gorilla in Kahuzi-Biega National Park and other sites

Line-transect data were collected by trained field teams walking 3-km transects that were established using the planning design module in Distance 6.0 [13]. Observers walked these lines silently at about 1-km h^-1^ to ensure that they were able to spot animals and record all sightings of primates, and ape nests and sign. In 1994, surveys were made in the Itebero, Lulingu and Nzovu sectors of KBNP. The same areas were surveyed between 2013 and 2015. We also carried out surveys in the Kasese Sector of the park in the west (in 2015), the Concession Forestière Communautaire des Banisamasi (CFCB) north of the park (in 2011), and the Tayna and Kisimba-Ikobo reserves (in 2013). A total transect length of 320.0 km was walked in 1994 and 277.7 km in the 2011–2015 surveys. We combined perpendicular distance to nest groups from the 1994 surveys with the more recent surveys and used the Multiple Covariate Distance Sampling (MCDS) analysis option in Distance 6.0 with survey period (1994 vs. 2011–2015) as a covariate. This option was used because the number of nest group sightings for the recent surveys was too few (16 nest groups) to estimate a separate detection function. MCDS constrains the shape of the curve to be similar across time periods but allows the scale of detection to vary. Perpendicular distances to nest groups were analysed rather than to individual nests [14] to be comparable to the analyses made in 1994. Nest density estimates were converted to densities of weaned gorillas assuming that each constructs one nest per day and average decay rate was 106 days—the method used in the 1994 analyses [5, 15].

#### Comparison of encounter rates

Encounter rate data were calculated from transect, recce and SMART datasets from 10 sites (protected areas or sectors within protected areas) where data existed for at least two time periods at the same site. A total distance of 12,730 km was walked at these sites between 1994 and 2015, with 977 km walked in the 1990s, 2,044 km between 2000 and 2010, and 9,709 km between 2011 and 2015. The number of gorilla nests per km walked (encounter rate) was calculated for each dataset for specific survey periods. The most recent encounter rates at a site were compared with the earliest encounter rate from the same site where the same method had been used (i.e. transect datasets were compared and recce datasets were compared, but because encounter rates tend to be higher on transects than on recces we did not compare between them). The percentage of the final encounter rate value to the initial value was calculated and the percentage loss was divided by the number of years between the two estimates to obtain a rate of loss per year.

#### Occupancy probability

Initially we planned to collect targeted occupancy data from 120 randomly-selected 5 x 5 km cells from a grid laid over Grauer’s gorilla’s known range. This grid size was chosen because it is approximately the size of gorilla home ranges in this region [12, 16, 17]. Insecurity in the region between 2012 and 2015 prevented teams from surveying more than 40 of these cells. We therefore used the full set of data from SMART, recces and transects, and combined them with data from the 40 cells surveyed in the occupancy analysis to accumulate a dataset of 1,061 sampled cells. Each dataset consisted of point data with GPS coordinates. The points were linked with lines for each patrol/survey to produce tracks of where teams had passed. These were then cut into cell-segments for each 5 x 5 km cell across the landscape to obtain the length of track in each cell. These cell-segments were then further divided and the presence of gorilla or chimp sign recorded for each 1-km unit in each cell. Only track lengths of 1 km were used (shorter sections were excluded). We used spatial replication of these 1-km segments in each 5 x 5 km grid cell for the occupancy analysis [18]. The number of units ranged from two to 50 per cell. Cells with zero gorilla sign were added in areas where we were certain gorillas were not present (lakes, agricultural land, settlements, and forest well beyond their known historical range).

The occupancy analysis was performed using the R-package hSDM [19]. This package uses a hierarchical Bayesian approach that can incorporate spatial dependency in the analysis. When estimating occupancy of the 5 x 5 km cells across the landscape, two issues needed to be considered: 1) imperfect detection and 2) spatial correlation. When a team visits a cell and walks through it they either detect sign of gorillas or they do not. If they do not, it may be because the gorillas were truly not there (true absence) or because the signs were missed but actually were there (false absence). Occupancy analysis enables an estimate of the detection probability to be made, which is usually less than 1 because some animals are missed in most surveys. A hierarchical or mixture model approach is used to estimate detectability, and Bayesian statistical methods used to estimate the parameters of such complex models. Taking spatial correlation into account is important to determine species range because most species show some form of geographical patchiness that can be explained by “hidden” biological (e.g., animals in groups) or environmental (e.g., geographical barriers) variables [19, 20]. We used covariables to predict from the sampled cells where we could calculate occupancy to estimate occupancy probabilities across the landscape. Thirteen covariables (see Table A in S1 File) were used to predict occupancy probability using the hSDM R package [21]. We used a combination of climate, topographic and human impact variables. Initially we correlated these variables and removed those that had a Pearson correlation coefficient greater than 0.7. Covariables were standardised by subtracting values from the mean and dividing by the standard deviation.

We ran the analyses using the hSDM Zero Inflated Binomial (ZIB) model [22], which assumes no difference in detectability between replicates. In this case, we were sampling 1-km replicate lines around the same time in each cell so it was unlikely that there would be a major difference in detectability. The hSDM.ZIB() function uses a mixture model that combines a Binomial process for observability and a Bernoulli process for habitat suitability. Effectively it fitted a logistic curve to the covariable layers to predict occupancy across the landscape. We ran the model with and without a spatial correlation analysis. The spatial correlation analysis incorporated an intrinsic Conditional Autoregressive model (iCAR), which assesses the spatial configuration of the eight nearest neighbouring cells to measure the spatial autocorrelation [20] (see Model A in S1 File).

We ran the hSDM.ZIB and hSDM.ZIB.iCAR models in the following manner:

1. Run an hSDM.ZIB model with environmental (climate and topographical) covariables
2. Select the significant variables from 1 and rerun hSDM.ZIB
3. Keep the significant variables from 2 and run hSDM.ZIB with human impact covariables also
4. Run hSDM.ZIB with variables from 2 and significant human impact covariables
5. Run hSDM.ZIB.iCAR with significant climate/topographical and human impact covariables and spatial auto-correlation.

This allowed us to assess the relative importance of each environmental and human impact variable on ape occupancy across the landscape. The effect of a variable was considered significant if zero was outside the 95% confidence interval of the parameter posterior distribution. For the parameter inference in a Bayesian framework, we used non-informative priors with large variance: Normal(mean = 0,variance = 10e^6^), except for the variance parameter of the spatial random effects, for which we used a weak informative prior: Uniform(min = 0,max = 10). We ran two parallel MCMCs for each parameter and checked the convergence of the chains visually and using the Gelman and Rubin’s convergence diagnostic.

#### Estimating Grauer’s gorilla numbers

To transform the map of probabilities of presence from the occupancy analysis into a gorilla distribution range we calculated a probability threshold maximizing the True Skill Statistic (TSS; Fig A in S1 File) [23]. Using data from nine sites where we had density estimates from line transect surveys in 1994 and 2011–2015, we regressed the encounter rate of individual nests with the density of gorillas and found a significant relationship (Fig B in S1 File). Using the regression equation obtained and the values of encounter rate obtained at the 10 sites where we had encounter rate data, we estimated average density of gorillas across their range, weighting by the area of each site. The surface area of the gorilla distribution range was multiplied by the weighted density of Grauer’s gorillas to estimate the total population size. Computing lower and upper confidence limits around this number, we need to take into account the uncertainty around density estimates as well as the uncertainty in the gorilla distribution range estimated from the hSDM.ZIB.iCAR model. Both sources of uncertainty were taken into account by combining the 95% quantiles (2.5% and 97.5% values) for both measures (area and density).

## Results

### Comparison of density estimates

Between 2011 and 2015 we surveyed the areas of KBNP covered in 1994–95 (Itebero and Nzovu sectors) and three other sites: the Kasese Sector, CFCB forest, and the Tayna and Kisimba-Ikobo reserves. When we calculated densities and estimated population numbers, we uncovered dramatic declines in nest and gorilla densities in the same sectors of KBNP (Table 1): the Itebero-Lulingu Sector (Zones 1–3 [5]) and Nzovu Sector (Zone 4 [5]). Combining the data for both sectors gives an 86.6% reduction in gorilla numbers for the lowland area of this park.

**Table 1:** Estimated density of gorillas at five sites from line-transect surveys.

Site	Area	Gorilla density 1994–95	Gorilla density 2011–15	Gorilla population 1994	Gorilla population 2011–15
KBNP Itebero-Lulingu	2,925	1.926	0.224	5,635 (2,995–10,633)	655 (206–2,134)
KBNP Nzovu	1,921	0.691	0.134	1,188 (605–2,343)	258 (86–811)
KBNP Kasese	716		0.256		183 (60–536)
CFCB	200		0.122		24 (4–147)
Tayna-Kisimba-Ikobo	1,869		0.289		541 (121–2,414)

The estimated density of gorillas calculated using standard line-transect survey analyses and using identical methods for datasets from 1994 and 2011–15. The estimated numbers of weaned individuals at five sites surveyed in 1994–95 and 2011–15 are given with 95% confidence limits of the population sizes in parentheses.

### Comparison of encounter rates

Comparisons of nest encounter rates (i.e. the number of nests counted per km walked on transects) showed major declines (81.7–100%) in six of the 10 sites: Balala Forest, Itombwe Reserve, KBNP (Itebero-Lulingu and Nzovu sectors), Maiko National Park, and RGPU north (Table 2). Smaller declines (5–10%) in encounter rates were observed in the Kasese Sector of KBNP, RGPU east and Usala Forest. In only one site, the highland Tshivanga Sector of KBNP, was an increase in encounter rates observed (following a drastic decline between 1994–2000). Half the population at Tshivanga was lost in the 1990s, when insecurity compromised the antipoaching activities of the park’s staff, but it has since recovered with improved protection. The steady growth of this population since 2000 is attributed to highly-targeted protection efforts by the protected area authority, the Institut Congolais pour la Conservation de la Nature (ICCN), together with rehabilitation of the gorilla tourism programme. Across the six sites exhibiting a major decline, the average rate of decline was 6.0% per year, with an average total decline of 94.2% over the entire range during the past 20 years (1994–2015).

**Table 2:** Changes in encounter rate at 10 sites across Grauer’s gorilla range.

Site	Dates of e-rate measurement	e-rate (first date)	e-rate (final date)	Percentage rate of decline per year	Percentage of originale-rate
KBNP Tshivanga	2000/2014	0.89	1.31	–5.4	147.2
KBNP Kasese	2013/2015	0.19	0.18	2.6	94.7
Usala Forest	2007/2014	1.47	1.37	1	93.1
RGPU east	1995/2014	0.72	0.65	0.5	90.3
RGPU north	1995/2014	0.93	0.17	4.3	18.3
KBNP Nzovu	1994/2014	1.21	0.11	4.6	9.1
KBNP Itebero-Lulingu	1994/2013	2.39	0.09	5.1	3.8
Itombwe Reserve	1996/2014	0.61	0.02	5.4	3.3
Maiko National Park	2005/2014	0.42	0.002	11.1	0.5
Balala Forest	1996/2014	0.17	0.00	5.9	0.0

The encounter rate (e-rate: number per km walked) of gorilla sign for different sites in Grauer’s gorilla’s range. The rate of decline in e-rate is calculated per year in addition to the total percentage change in e-rate at each site.

### Occupancy Analysis

Three significant environmental variables were identified (distance to active deforestation (m); elevation (m), and tree cover (%)) which together explained 17% of the model fit, and the spatial correlation explained a further 39% (Table 3). Grauer’s gorilla is, therefore, found in high altitude areas far from areas where people are clearing forest, with a high tree cover; effectively places people tend not to be.

**Table 3:** Model results comparing deviance values and percentage of deviance explained.

Model	Deviance	Percentage of deviance explained	Covariables
1 NULL	1126.6	0	Null model with mean parameter and no covariables
2 environment	1048.1	13	Tree cover and elevation
3 env+human impact	1029.3	17	Adding distance to forest loss
4 env+hum+iCAR	798.8	56	Adding in iCAR
5 FULL	541.3	100	Full model with as many parameters as observations

**Fig 1:**
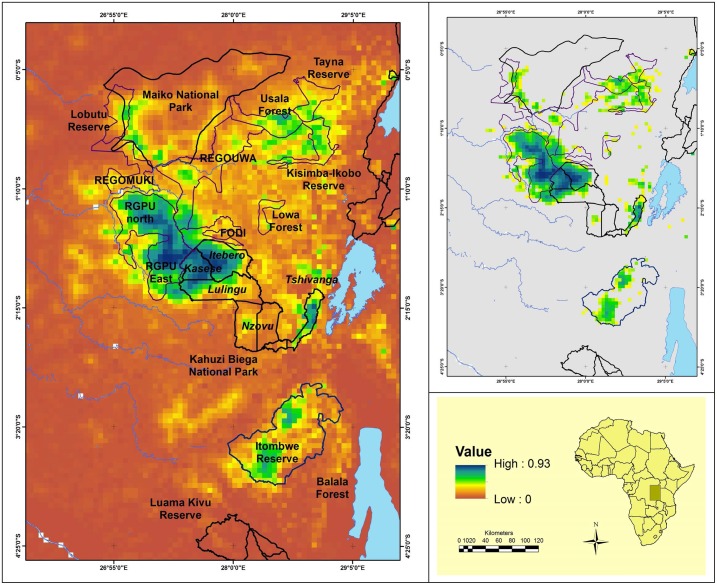
Map of the probability of presence for Grauer’s gorilla. Occupancy probability was mapped using three covariables (tree cover, altitude and distance to recent deforestation) and a spatial autocorrelation (left panel). The probability threshold maximising the TSS was 0.35. This threshold was used to derive a gorilla distribution range (upper right panel) for which presence probabilities were greater than this value (grey = less than 0.35).

Occupancy analysis, incorporating this spatial autocorrelation (using an intrinsic Conditional Autoregressive Model), and using these three significant predictor covariables in a Bayesian framework (see methods) showed that KBNP, RGPU and Usala forest remain critical sites for Grauer’s gorilla conservation (Fig 1). However, the extent of the KBNP-RGPU region where gorillas are present (Fig 1) has decreased in size from previous estimates of 15,870 in 1959 [5, 24] to 12,770 km^2^ in 1994 [5] to 9,005 km^2^ today, a 29.5% loss in surface area since 1994 and 43.3% loss since 1959. Other areas of importance for Grauer’s populations are parts of the Itombwe Reserve, the Tayna and Kisimba-Ikobo community reserves.

We computed the probability threshold of the occupancy data (0.35), maximizing the TSS to estimate the area where gorillas are likely to occur. The area of habitat where the occupancy probability exceeded the threshold was 19,700 km^2^. A regression of gorilla density on nest encounter rates using data from nine sites was significant (R^2^a_dj_ = 0.96 –see Fig B in S1 File) and allowed us to predict gorilla density at the sites for which we had encounter rate data. The mean density of gorillas across their range was 0.193 km^-2^, weighted by the area of the site. We then estimated the number of Grauer’s gorillas remaining in the wild by multiplying this area by the mean density and produced an estimate of 3,800 individuals.

#### Upper and lower 95% confidence interval maps

**Fig 2:**
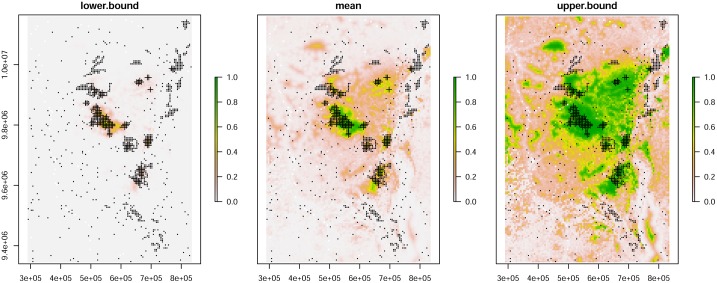
The mean (centre) and 95% confidence interval (left: lower bound; right: upper bound) of the probability of presence for Grauer’s gorilla. The maps also show the location of cells sampled (grey dots) with cells where gorilla sign was observed (black crosses).

We computed the 95% confidence interval of the posterior distribution of the probability of presence for each spatial cell using argument save.p = 1 in the hSDM R package. We used this confidence interval to generate an upper and lower confidence probability map (Fig 2).

**Fig 3:**
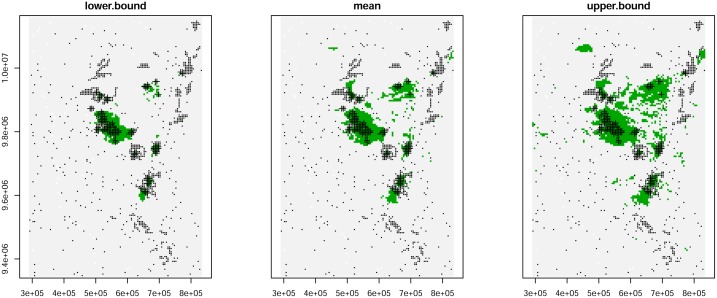
The mean (centre) and 95% confidence interval (left: lower bound; right upper bound) of the gorilla distribution range using the same boundaries as Fig 2.

For the three maps of probability, we computed the probability threshold maximizing the TSS and derived the mean and 95% confidence interval for the gorilla distribution range (Fig 3).

Lower and upper confidence limits around the estimated numbers of gorillas across their range were computed from the uncertainty in the regression of encounter rate against density as well as the uncertainty in the gorilla distribution range resulting from the hSDM.ZIB.iCAR model. Combining both sources of uncertainty, the estimate of Grauer’s gorilla numbers is 3,800 (95% CL: 1,280–9,050).

## Discussion

### Survey coverage and gorilla densities

This survey of Grauer’s gorilla is the most extensive ever made for this ape [4, 6]. More sites were visited than have been previously to obtain quantitative data and more than 12,000 km were walked to obtain the data. Field teams conducted intensive surveys even in regions of insecurity where access was possible, searching for ground nests and other signs of this elusive ape. Insecurity has cost the lives of more than 150 ICCN rangers in eastern DRC in the past 20 years. Collecting data on Grauer’s gorillas is therefore fraught with difficulty. WCS has established SMART databases across much of eastern DRC, in both national parks and community reserves. Rangers and ecoguards now have the ability to collect data on illegal activities and record wildlife observations on their smartphones during patrols. We used data collected by local community members and ICCN rangers, entered into SMART, because this enabled data to be collated from across most of Grauer’s gorilla’s range. SMART data are not collected uniformly across an area, but by analysing occupancy in grid cells and employing the novel approach of incorporating spatial autocorrelation in a Bayesian framework, it is possible to estimate the occupancy probability in a rigorous manner.

Quantitative surveys of great apes are time consuming and expensive, involving line transects and ideally the measurement of decay rates of nests in the region being surveyed. Decay rates are often borrowed from other sites because of these costs or lack of time [15]. The tight regression we obtained between encounter rate of numbers of individual nests along transects and the density of gorillas at a site (R^2^ = 0.96) means that encounter rates, which are easier and less costly to obtain, could be used to estimate gorilla density at sites across Grauer’s gorilla range in future.

#### Hierarchical Bayesian species distribution model to improve species range estimate

The approach used here to calculate occupancy, incorporating spatial autocorrelation, converting this to a threshold value using the TSS, and weighting average density when calculating gorilla numbers is a novel way of estimating great ape population size, and one that will allow estimates to be made for other apes and elusive species.

Of the 13 environmental and human-activity related variables initially tested to explain the distribution of the Grauer’s gorilla, we selected only three explanatory variables based on statistical significance and biological coherence: tree cover, altitude and distance to recent deforestation. While the effects of these variables were easy to interpret (gorillas are most likely found in remote access areas at high altitude and under closed canopy forest, far away from recent human disturbance), they explained only 17% of the null model deviance (Table 3). Using only these variables in the species distribution model, we would have had a rough estimate of the potential suitable habitat for the gorillas, but it would not have been possible to estimate the current species range with precision [25]. Including spatial random effects through the iCAR process, we were able to explain up to 56% of the null model deviance, increasing the deviance explained by 39 points. The final model had a high TSS value of 0.87 and was thus able to predict accurately both gorilla presence and absence. The resulting distribution map, albeit imperfect and showing some false-presence sites (e.g., Rwenzori National Park), was coherent in comparison with observational data and expert knowledge [26].

Many species show some form of geographical patchiness [19, 20], as we have found for Grauer’s gorilla. Including spatial autocorrelation in species distribution models accounts for the effects of “hidden” variables that explain patchiness in their distribution [19]. These hidden variables can be intrinsic biological variables (e.g., social animals living in groups) or extrinsic environmental variables (e.g., unavailable climatic variables such as air humidity, or geographic barriers impeding species dispersion). Software packages commonly used to model species distribution, such as Maxent [27] or Biomod [28], do not account explicitly for spatial-autocorrelation. Here, we show the advantage of using a hierarchical Bayesian species distribution model with spatial autocorrelation to estimate a species' range more accurately. This was made possible by the hSDM R package, which provides functions for estimating the parameters of an iCAR process. This approach can be easily extended to other species showing geographical patchiness and for which access to significant explanatory variables is limited (see [29, 30] for fish and bird examples, respectively).

Another advantage of our hierarchical model compared to the classical approaches cited above is the possibility of accounting for false-absence sites through the estimation of a detection parameter. Finding traces of gorilla presence (direct detection of individuals, or indirect signs of presence such as nests or faeces) in dense tropical forest is not easy. Not accounting for imperfect detection can drastically reduce the performance of species distribution models [31]. To account for imperfect detection, we used a zero-inflated binomial (ZIB) model combining a Binomial distribution for the suitability process and a Bernoulli distribution for the detectability process. We estimated an 11% probability of detecting gorilla presence if the site was suitable. Accounting for imperfect detection, we aimed at increasing the performance of our model to provide more accurate estimates of the species' range and population size.

### Conservation status of Grauer’s gorilla

#### Updating the conservation status of Grauer’s gorilla to Critically Endangered

It was suspected that a large decline in Grauer’s gorilla numbers was likely because of the extensive insecurity and evidence of poaching of apes by armed militias and rebel groups [1, 32]. Our results show there has been a mean reduction of 87% in gorilla density in the lowland area of KBNP, an average decline of 94% in nest encounter rates at six of 10 sites, and overall a 77% decline in the estimated total number of individuals across its range. It is likely that our model tended to overestimate the area (give false presences rather than false absences [21]), therefore the 77% decline is a minimum estimate. In order for a taxon to be listed as Critically Endangered on the IUCN Red List it must decline by more than 80% over three generations [33]. Gorillas can live for 30–40 years, but the average generation time of Grauer’s gorillas is 20 years [1]. Our results show at least a 77% decline in Grauer’s gorilla in just one generation, which qualifies the taxon as Critically Endangered (CR A4bcd). Mountain gorillas are already listed as Critically Endangered [34] and a similar listing for Grauer’s gorilla will classify the eastern gorilla species as Critically Endangered, with a population size of fewer than 5,000 individuals. This uplisting will categorise all gorilla taxa as Critically Endangered.

#### Conservation actions for protecting Grauer’s gorilla populations

Halting and even reversing the decline of Grauer’s gorilla will take considerable effort and will require more funding than is currently available. Artisanal mining must be regulated, and the various rebel groups controlling the mines disarmed. To accomplish this, it will be necessary to halt mining in protected areas, because miners subsist on bushmeat and hunt gorillas around their camps [11]. We urge the Government of DRC to actively seek to control this part of the country for the benefit of both humans and gorillas. Significant efforts must be made for the government to regain control of this region of DRC. In particular, this should include professionalising the DRC military and increasing their pay, so that they are better motivated to collaborate with ICCN and partners to protect the wildlife of this region. Grauer’s gorilla is endemic to DRC and a symbol of the Albertine Rift, one of the richest areas on the planet for biodiversity. Unless significantly greater investment is made to conserve them, we are likely to lose Grauer’s gorilla from many sites across its range in the next five years.

## Supporting Information

S1 File Supporting Tables, figures and details of model used. (DOCX)
