# Supplementary material for: Catastrophic Decline of World's Largest Primate: 80% Loss of Grauer's Gorilla (Gorilla beringei graueri) Population Justifies Critically Endangered Status
Source: PLoS One. 2016 Oct 19;11(10):e0162697. doi: 10.1371/journal.pone.0162697 (PMC5070872; doi:10.1371/journal.pone.0162697)
Supplement: S1 File — (DOCX) [file pone.0162697.s001.docx]

**Supporting Information: Grauer’s gorilla population crash**

**Table A.** Covariables used for the analysis of occupancy across the landscape.

| **Covariable Name** | **Measures** | **Source** |
| --- | --- | --- |
| *Climate variables* | | |
| bio2 | Mean diurnal temperature range | WorldClim interpolated climate surfaces  <http://www.worldclim.org/> |
| bio12 | Mean annual precipitation |  |
| bio17 | Precipitation of driest quarter |  |
| *Topographic and forest variables* | | |
| dem | Elevation above sea level | SRTM data at University of Maryland  <http://glcf.umd.edu/data/srtm/> |
| rugged | Ruggedness of topography | Available at <http://diegopuga.org/data/rugged/#grid> |
| slope | Slope – calculated from DEM layer | SRTM data at University of Maryland  <http://glcf.umd.edu/data/srtm/> |
| stslopdis | Distance to steep slopes | Calculated by Lilian Pintea, Jane Goodall Institute, from SRTM data |
| treecov | Percentage tree cover | Calculated by Lilian Pintea, Jane Goodall Institute, from Hansen *et al*. (2013)* |
| *Human impact variables* | | |
| disforlos | Distance to forest recently lost | Calculated by Lilian Pintea, Jane Goodall Institute, from Hansen *et al.* (2013)* |
| minedist | Distance to artisanal mines | Data from International Peace Information Service and mine location data from SMART |
| rivdis | Distance to rivers | Calculated from |
| roaddis | Distance to roads | Data from UNOCHA in eastern DRC |
| villdis | Distance to villages | Date from UNOCHA in eastern DRC |

*M.C. Hansen et al., High-resolution global maps of 21st-century forest cover change. Science 342: 850–53 (2013). Data available online from: <http://earthenginepartners.appspot.com/science-2013-global-forest>

**Model A: Model for occupancy analysis:**

The occupancy analysis was performed using a zero-inflated binomial model with spatial autocorrelation (hSDM.ZIB.iCAR() function in the hSDM R package^15^). This model is hierarchical and structured as follows:

There is an ***ecological process*** – the suitability of the habitat:

*z_i_ ~ Bernoulli (Ɵ_i_)*

*Logit (Ɵ_i_) = X_i_B + p_i_*

Where *z_i_* = habitat suitability at site *i*; *Ɵ_i_* = probability that habitat is suitable at site *i*. Habitat at site *i* is described by environmental variables *X_i_* with coefficients *B* and spatial random effect *p_i_*. *p_i_* is the spatial random effect for cell *i*.

Secondly, there is a ***spatial auto-correlation*** component:

An intrinsic conditional autoregressive model (iCAR) is assumed:

*p_i_= Normal (u_i_, V_p_/n_i_)*

Where *u_i_* = mean of *p_i_* in the neighbourhood of cell *i*; *V_p_* = variance of the spatial random effects; *n_i_*= number of neighbours for cell *i*

Thirdly, there is an **observation process**:

*y_i_ = Binomial (z_i_ * d_i_,t_i_)*

*Logit (d_i_) = W_i_ϒ*

Where *y_i_* = presence of a species at site *i*; *d_i_* = probability of detecting the species at site *i*; *W_i_* = covariables explaining the observation process with parameters *ϒ*. *t_i_* is the number of visits (trials) at site *i.*

Non-informative normal priors were used for parameters B and ϒ (mean=0 and variance=10^6^). For the variance of the spatial random effects (V_p_), we used an informative uniform prior on the interval [0,10].

***Gorilla model***

In the final hSDM.ZIB.iCAR model for gorillas, the following coefficients for each covariable that was statistically significant and biologically relevant were obtained:

| **Covariable** | **Mean** | **SD** | **Significance** |
| --- | --- | --- | --- |
| beta.(Intercept) | -4.074 | 0.449 | P<0.05 |
| beta.dem | 1.237 | 0.313 | P<0.05 |
| beta.disforlos | 1.233 | 0.419 | P<0.05 |
| beta.treecov | 0.794 | 0.243 | P<0.05 |
| gamma.(Intercept) | -2.082 | 0.068 | P<0.05 |
| Vrho | 9.391 | 0.563 |  |
| Deviance | 798.779 | 15.572 |  |
|  |  |  |  |

Gorillas tend to be found at higher elevations, where tree cover is high, and away from active deforestation. Posterior mean detection probability (*d_i_*) was 0.111 and the posterior mean probability of occupancy (*Ɵ_i_*) was 0.073.

**TSS: Computation of TSS and gorilla numbers**

We obtained a probability threshold of 0.35 (Fig A) and a maximal TSS of 0.88. This is a relatively high TSS value, indicating a good correspondence between our species distribution area and observed suitable and unsuitable sites. The species distribution area, defined as the 5x5 km cells with a presence probability value of 0.35 or greater, was 19,700 km^2^. The weighted mean density of gorillas across all sites, applying the regression of encounter rate on density (Fig B) to sites with encounter rate data, was 0.193 per km^2^. When multiplied by the surface of the species distribution area, we estimated a total population of 3,800 Grauer’s gorillas across the range.


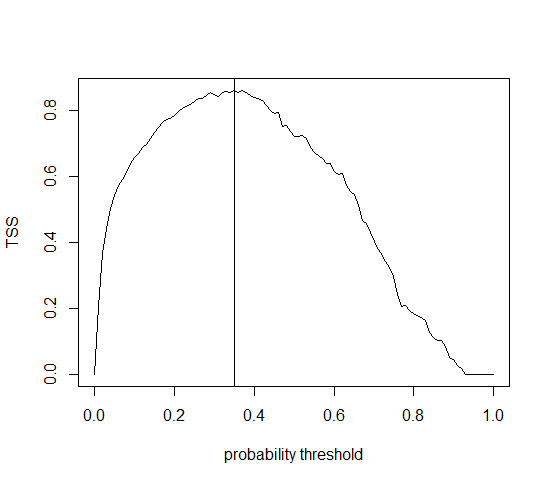


**Figure A.** Plot of the True Skill Statistic against probability threshold identifying p=0.35 as the probability threshold for a maximum TSS of 0.88.


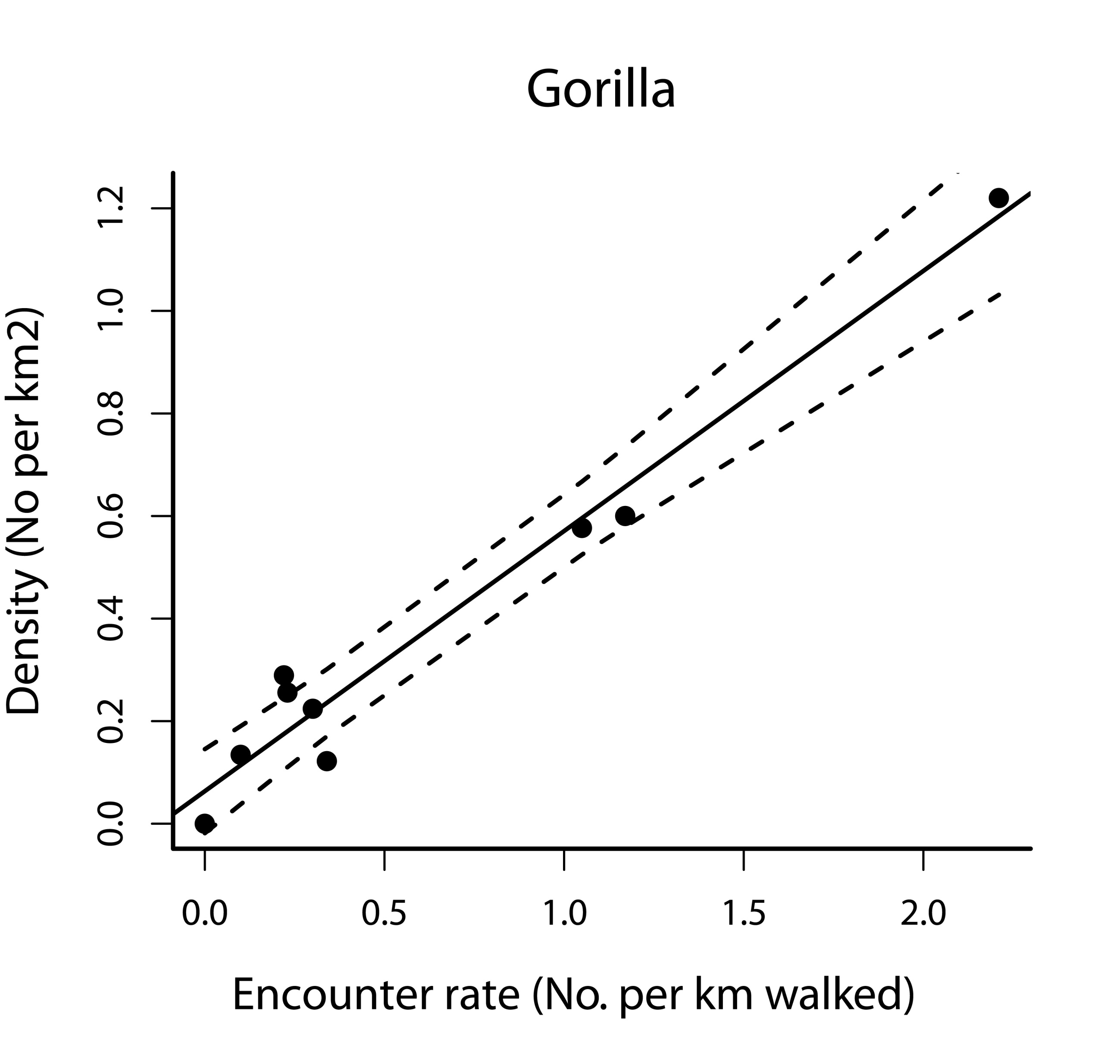


**Figure B.** Correlation of encounter rate of nests (No. per km walked) and calculated densities (No. per km^2^) with 95% confidence intervals (dashed lines). Density = 0.507 x e-rate +0.064 (R^2^_adj_=0.96). Densities were calculated for nine sites for which transect data were available.

**R-Code**

A project has been established at GitHub, which is open access: <https://github.com/ghislainv/gorillas>
